# Influence of wettability and surface design on the adhesion of terrestrial cyanobacteria to additive manufactured biocarriers

**DOI:** 10.1007/s00449-022-02712-0

**Published:** 2022-03-02

**Authors:** Kai Scherer, Winda Soerjawinata, Susanne Schaefer, Isabelle Kockler, Roland Ulber, Michael Lakatos, Ulrich Bröckel, Percy Kampeis, Michael Wahl

**Affiliations:** 1 Department of Environmental Planning and Technology, Environmental Campus Birkenfeld, Trier University of Applied Sciences, Campusallee, 55768 Hoppstädten-Weiersbach, Germany; 2 Institute of Bioprocess Engineering, Technical University of Kaiserslautern, Gottlieb-Daimler-Straße 49, 67663 Kaiserslautern, Germany; 3 Department of Applied Logistics and Polymer Sciences, University of Applied Sciences Kaiserslautern, Carl-Schurz Str. 10–16, 66953 Pirmasens, Germany

**Keywords:** Additive manufacturing, Biocarriers, Biofilm, Photobioreactor, Terrestrial cyanobacteria

## Abstract

Productive biofilms are gaining growing interest in research due to their potential of producing valuable compounds and bioactive substances such as antibiotics. This is supported by recent developments in biofilm photobioreactors that established the controlled phototrophic cultivation of algae and cyanobacteria. Cultivation of biofilms can be challenging due to the need of surfaces for biofilm adhesion. The total production of biomass, and thus production of e.g. bioactive substances, within the bioreactor volume highly depends on the available cultivation surface. To achieve an enlargement of surface area for biofilm photobioreactors, biocarriers can be implemented in the cultivation. Thereby, material properties and design of the biocarriers are important for initial biofilm formation and growth of cyanobacteria. In this study, special biocarriers were designed and additively manufactured to investigate different polymeric materials and surface designs regarding biofilm adhesion of the terrestrial cyanobacterium *Nostoc flagelliforme* (CCAP 1453/33). Properties of 3D-printed materials were characterized by determination of wettability, surface roughness, and density. To evaluate the influence of wettability on biofilm formation, material properties were specifically modified by gas-phase fluorination and biofilm formation was analyzed on biocarriers with basic and optimized geometry in shaking flask cultivation. We found that different polymeric materials revealed no significant differences in wettability and with identical surface design no significant effect on biomass adhesion was observed. However, materials treated with fluorination as well as optimized biocarrier design showed improved wettability and an increase in biomass adhesion per biocarrier surface.

## Introduction

Phototrophic biofilms composed of terrestrial cyanobacteria are embedded in a matrix of extracellular polymeric substances (EPS) [1]. The EPS contain various polysaccharides, lipids, and extracellular proteins that lead to a stable production environment, enhanced mechanical stability, and increased surface adhesion [2, 3]. Due to the discovery of several valuable compounds with antibacterial and antifungal activities [4, 5] in biomass and EPS [6–8], cyanobacteria are beneficial to the development of drugs such as antibiotics and offer a great potential for pharmaceutical applications [9, 10].

To use this potential and to enable an industrial grade of utilization, the optimization of production technologies for cyanobacteria is necessary. For the controlled phototrophic cultivation of cyanobacteria and production of bioactive substances, special reactors, called photobioreactors, are used. Over the past few decades, their operation and technology have been progressively improved and aspects such as light distribution [11, 12] or temperature control [13, 14] have been examined. Most cultivation systems are designed for aquatic cyanobacteria and therefore a submerged cultivation with liquid media as suspension. For terrestrial cyanobacteria, originated from emersed air-exposed habitats, however, the cultivation conditions in submerged fermentations are not optimal and their productivity is limited [15, 16]. To address these limitations, a special biofilm cultivation system, the emersed photobioreactor (ePBR), was developed by Kuhne et al. [17] and steadily refined over the past few years [18–20]. The ePBR imitates the natural habitat of terrestrial cyanobacteria by an air exposed cultivation and nutrient supply through an aerosol. The aerosol-based cultivation results in reduced water consumption and allows better process control, for example, the aimed desiccation to induce the production of EPS [21]. Surface attached cultivation of cyanobacteria in biofilm photobioreactors led to higher biomass productivity and more efficient harvesting processes compared to suspended cultivations [18, 22, 23]. When cultivated as biofilms, the biomass production depends on the available cultivation surface. To maximize this cultivation surface and to keep the volumetric requirements low, regular shaped biocarriers can be used as a substrate for surface attached biofilm growth.

Biocarriers and packings are designed to increase the reactive surface area for biochemical, chemical, or thermal processes. Therefore, they are already implemented in several industrial applications. In thermal process engineering, for example, packings are used as packed columns for heat and mass transfer processes in rectification, absorption, and extraction [24]. Heterotrophic biofilm reactors in wastewater treatment use biocarriers to provide cultivation systems with decreased volume requirement, consistent production, and enhanced mass transfer [25, 26]. Related to the variety of different application fields, biocarriers and packings are available in various sizes, geometries, and materials. Biocarriers must fulfill specific requirements for the application in photobioreactors. A high specific surface is required to increase the surface–volume ratio and optimize volumetric productivity. Due to the phototrophic cultivation, light supply is an important factor, which is affected by geometry, porosity, and material transparency. Flow characteristics are also crucial as they influence the mixing efficiency in submerged and the aerosol distribution in emerged fermentations. Furthermore, the choice of biocarrier material regarding performance (e.g. toxicity, flexibility), the material and media composition (e.g. pH value) and surface properties such as roughness and wettability are essential for initial biofilm adhesion [27], growth, and harvesting. However, not only the biocarrier material, but also the manufacturing process plays a key role.

Additive manufacturing (AM), commonly known as 3D-printing, is a rapid evolving manufacturing technique. Due to the variety of inexpensive print materials and 3D-printers, fused filament fabrication (FFF) is one of the most common AM processes. A polymeric filament is used in FFF to generate a solid model, layer by layer, on a build platform. Single extruded lines are combined to a slice of the model in each layer. Thereby, material is extruded through a heated nozzle with a cylindrical outlet, resulting in lines with circular cross-section. These lines are placed close to each other and fused together, but their circularity remains noticeable on the surface of the model and especially on the top layer. Orientation of the top layer lines can be influenced by model placement on the printer and presets in the slicing software. Due to the circular extrusion, the FFF process leaves small grooves between each extruded line vertically and horizontally. Another AM process is digital light processing (DLP), which manufactures solid models out of liquid photopolymers. In DLP 3D-printing, an image of one layer of the model is projected to a build platform and the photopolymer is cured in designated zones using photopolymerization. During printing, photopolymer is supplied in a resin tank and models are built layer by layer to the build platform. There are no grooves on the surface and overall finish is smooth because the projected image consists of square pixels. AM has already been used to investigate biocarrier geometry [28] and adhesion of aquatic microalgae and macroalgae to different surface topographies [29]. However, there is no literature available regarding biofilm adhesion of terrestrial cyanobacteria to additively manufactured biocarriers.

This study focused on the impact of biocarriers’ surface and material characteristics for biofilm adhesion of terrestrial cyanobacteria to optimize emerged and submerged fermentations. AM was used to manufacture polymeric biocarriers and material samples for contact angle and surface roughness measurements. To specify the influence of wettability for biofilm adhesion, surface characteristics of 3D-printed materials were altered by gas-phase fluorination. In addition, terrestrial cyanobacteria were cultivated on suspended biocarriers by shaking flask cultivation to investigate biofilm formation and attachment on different polymeric biocarriers.

## Materials and methods

### Design of biocarriers

**Fig. 1:**
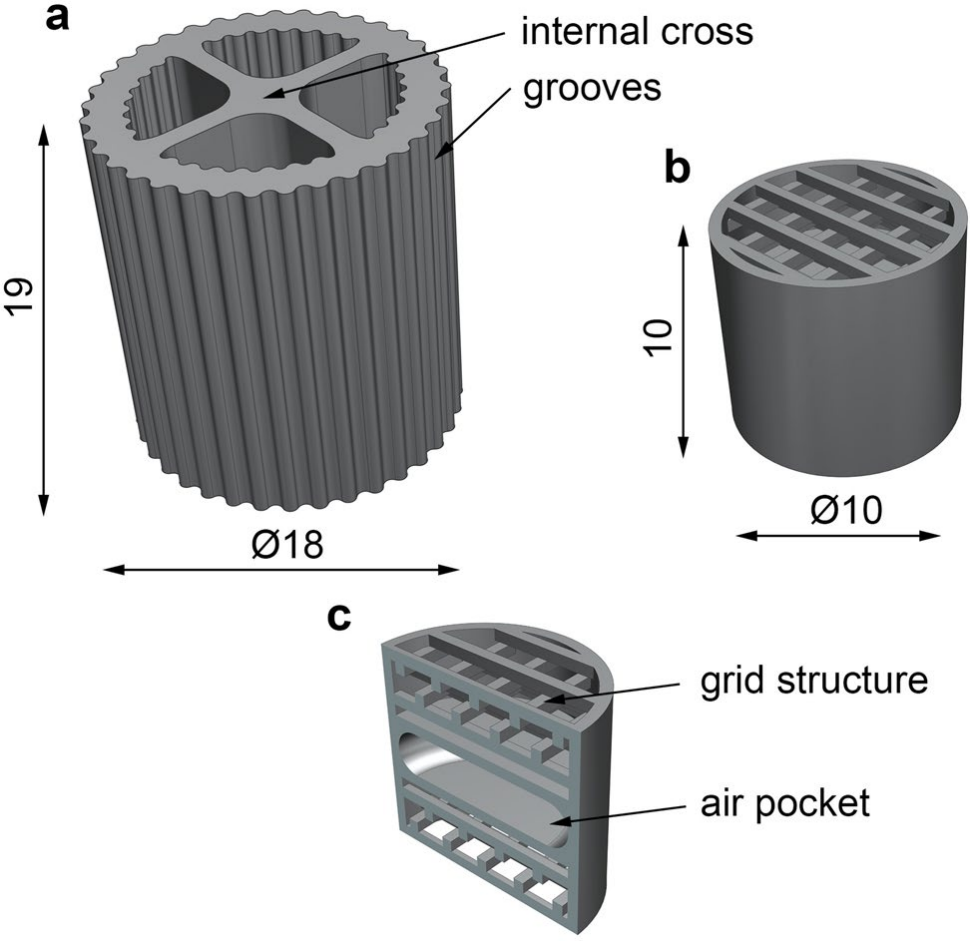
Geometry and dimensions of cultivated biocarriers. **a** FFF biocarrier, **b** optimized biocarrier and **c** inner structure and air pocket of optimized biocarrier

For the investigation of biofilm attachment, special biocarriers were designed in the computer aided design software (CAD) Siemens NX (Version 1859, Siemens PLM, Plano, USA). A simple biocarrier, intended for FFF 3D-printing (Fig. 1a), had an internal cross and grooves on the inner and outer shell to enhance mechanical stability and increase specific surface. The biocarrier was cylindrical shaped, 18 mm in diameter, 19 mm in height, and had a theoretical surface of 32 cm^2^ (determined in CAD).

**Fig. 2:**
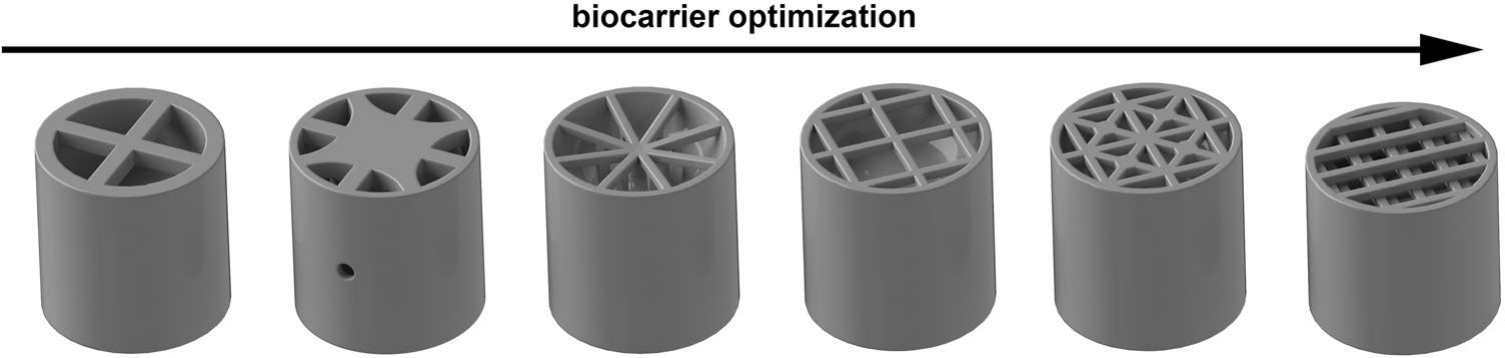
Design variations for the consecutive optimization of biocarrier geometry. Optimization is displayed from left to right

Another biocarrier was designed to validate the influence of biocarrier geometry. The biocarrier was iteratively optimized from a hollow cylinder with internal crosses to a cylinder with a grid structure. To reach optimal biofilm attachment, different grid structures (Fig. 2) were tested in heterotrophic cultivation (data not shown) to optimize the retention of biomass. The optimized biocarrier (Fig. 1b) was 10 mm in diameter and height and had a theoretical surface of 12 cm^2^ (determined in CAD). The biocarrier was designed with an elliptical air pocket in the middle (Fig. 1c) to achieve a density of approximately 1 g cm^−3^ and attain suspension in the cultivation. An air pocket volume of 120 mm^3^ was used to achieve the desired biocarrier density. The grid structure contained four superimposed, displaced grids, each consisting of 0.5 mm thick rods with 1 mm spacing, to provide sufficient retention and support biofilm adhesion during cultivation.

### Manufacturing of biocarriers and material samples

The FFF biocarriers (Fig. 1a) were additively manufactured out of acrylonitrile butadiene styrene (ABS), polyethylene terephthalate glycol (PETG), polylactic acid (PLA), and polypropylene (PP) using a Ultimaker 3 (Ultimaker B.V., Cambridge, USA) with a 0.4 mm nozzle. All models for FFF printing were sliced with Ultimaker Cura (Version 4.8.0, Ultimaker B.V., Cambridge, USA). Extruder and build plate temperature during fabrication were set to 230 °C and 95 °C for ABS, 240 °C and 95 °C for PETG, 220 °C and 60 °C for PLA, and 210 °C and 95 °C for PP, respectively. All filaments were 2.85 mm in diameter and produced by innofil3d (BASF 3D Printing Solutions, Emmen, Netherlands). Biocarriers were printed at a layer height of 0.25 mm using only wall lines to generate a solid model. All carriers were marked with a defined dot pattern on the top and bottom side with a soldering iron to allow clear identification.

Due to the grid structures, optimized biocarriers (Fig. 1b) were fabricated in a different additive manufacturing process out of polyacrylate (PAR). E-Shell 600 clear (DeltaMed GmbH, Friedberg, Germany) was used to manufacture PAR biocarriers with a Vida 3D-printer (EnvisionTEC GmbH, Gladbeck, Germany) using the principle of inverse DLP at a temperature of 23 °C. The light projector has a power of 330 W and a pixel width of 73 × 73 µm with a resolution of 1920 × 1080 pixels. E-Shell 600 clear is suitable for pharmaceutical applications because it is United States Pharmacopeia Class VI certified and thus biocompatible according to ISO 10993. Support structures were added with Materialise Magics (Version 20.2.0.24, Materialise NV, Munich, Germany) to ensure stability during the printing process. The part with supports was divided into individual layers with the Perfactory Rapid Prototyping software (Version 3.2.3377.1712, EnvisionTEC GmbH, Gladbeck, Germany), corresponding to the selected layer height of 50 µm in the 3D-printing process.

**Fig. 3:**
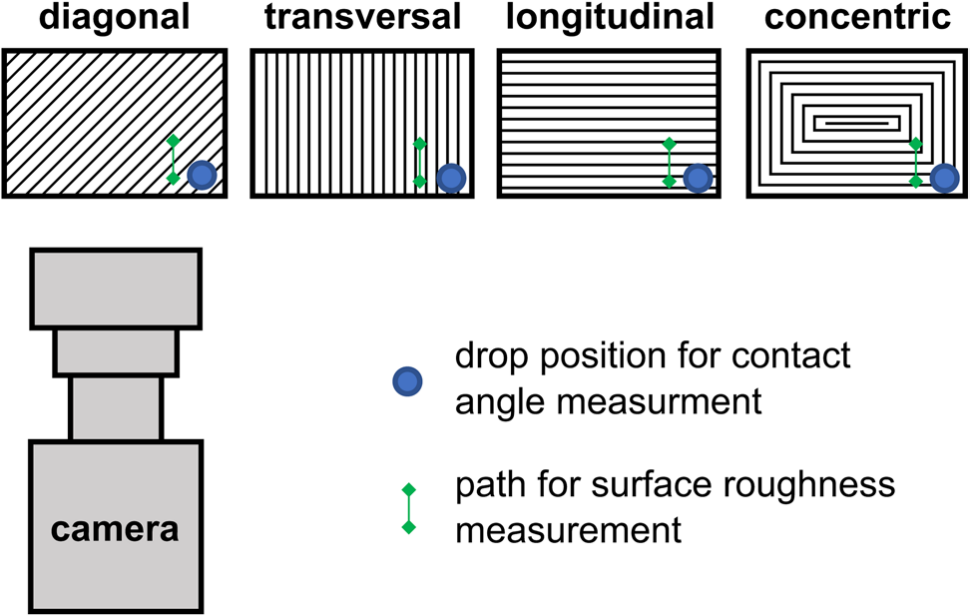
Top view of the experimental setup for contact angle and surface roughness measurements showing the placement of the measurements in relation to the top layer orientation of the 3D-printed samples (not to scale)

For the characterization of contact angle and surface roughness, rectangular samples (48 mm long, 28 mm wide and 1 mm thick) were fabricated by FFF 3D-printing (Ultimaker 3, Ultimaker B.V., Cambridge, USA) at a layer height of 0.25 mm with 100% rectilinear infill out of ABS, PETG, PLA, and PP. Extruder and build plate temperature were equal to the temperatures chosen for printing the FFF biocarriers. The lines on the top layer of the samples were set to be either diagonal, transversal, longitudinal, or concentric (Fig. 3). The different top layer orientations for the surface samples were chosen because a mix of these line orientations resulted on the surfaces and outer shell of FFF printed biocarriers.

### Fluorination

After manufacturing, selected samples for surface characterization out of ABS, PETG, PLA, and PP as well as biocarriers out of PLA, PP, and PAR were fluorinated. Gas-phase fluorination alters the surface characteristics by substitution of hydrogen atoms with fluorine atoms on the polymer surface. Thereby, surfaces become more hydrophilic and wettability is increased. Fluorination was carried out by the CH Oberflächenservice GmbH (Dernbach, Germany) using a fluorine nitrogen gas mixture with 10 vol % fluorine at 25 °C.

### Material density

Density of the biocarriers was determined using a pycnometer (50 ml Duran, Schott AG, Mainz, Germany). The weighing was carried out with a precision balance (Sartorius ED224S Extend ED, Sartorius AG, Göttingen, Germany) to determine the displaced volume by the biocarrier and thus calculate the density. Density values for water were taken in relation to the water temperature.

### Contact angle and surface roughness measurement

Contact angle was measured to determine wettability of the manufactured samples using sessile drop method. The contact angle is defined by the angle between the tangent from the contact point at the surface to the liquid–gas phase and the horizontal surface on which the drop rests. Each measurement consisted of three replicates with 50 µL droplets. Instead of water, the standard growth medium of cyanobacteria BG 11 [30] was used, which is a nutrient deficient medium but in comparison to water containing a slightly higher mineral content. The drops were set to the surface of untreated and fluorinated samples (Fig. 3) and 30 pictures were taken with a high-speed camera (VW-600 M, Keyence Deutschland GmbH, Neu-Isenburg, Germany) accordingly. Contact angle was determined manually using the open-source image processing software ImageJ with the contact angle plugin and ellipse approximation.

Surface roughness was measured tactile (Perthometer S2, Mahr GmbH, Göttingen, Germany) and values for arithmetical mean deviation were exported with MarWin (Version 11.20–03 SP1, Mahr GmbH, Göttingen, Germany). Surface roughness was recorded with three replicates for each sample on a length of 4 mm parallel to the camera view in contact angle measurements (Fig. 3).

### Cultivation

The terrestrial cyanobacterium *Nostoc flagelliforme* (CCAP 1453/33) was used in the cultivations. Precultures were prepared by cultivating in 300 mL shaking flasks without baffles with 100 mL BG 11 at room temperature, a light intensity of approximately 20 µmol_photons_ m^−2^ s^−1^ photosynthetic photon flux density (PPFD), a light/dark rhythm of 12/12 h, and without shaking. The measurement of PPFD was performed using a quantum sensor (SQ-520, apogee instruments, Logan, USA). After 6 weeks of growth, the cells were harvested by centrifugation (1970 g, 10 min, room temperature).

The main cultivation was inoculated with 600 mg biomass wet weight (BWW) of precultures in 100 mL BG 11 medium per flask, corresponding to 59 mg biomass dry weight (BDW), which resulted in a culture with 590 mg_BDW_ L^−1^. Cultivation conditions were similar to the precultures with addition of 10 pre-dried and pre-weighted biocarriers exposed to a mean of 11.8 mg BDW per carrier. The 10 biocarriers of each material and treatment were distributed in two 100 mL flasks with 5 biocarriers of the same sort per flask. All carriers were sterilized using 70% ethanol under a sterile bench prior to the cultivation. The cultivation on carriers was conducted for 3 weeks without shaking.

At the end of the fermentation, overgrown biocarriers with *N. flagelliforme* (CCAP 1453/33) were harvested and put in an oven at 60 °C until constant weight was reached. BDW was determined by subtraction of pre-dried biocarrier weight from dry weight of cultivated biocarrier (biocarrier and biomass). BDW was expressed in milligram of dry weight per surface of biocarrier (mg_BDW_ m^−2^). After removal of the cultivated biocarriers, the BDW of remaining biomass in suspension was determined by two 10 mL samples per flask.

### Statistical analysis

Results were statistically analyzed for normality by Kolmogorov–Smirnov test and by analysis of variance (ANOVA) with post hoc test according to Fisher-LSD (least significant difference) for significant differences using Origin 2021 (OriginLab Corporation, Northampton, USA).

## Results

### Density of biocarriers

The hydrostatic behavior of the biocarriers was analyzed as biocarrier density. Measurement of density revealed almost identical values of 1.182 ± 0.001 g cm^−3^ for PLA and 1.188 ± 0.015 g cm^−3^ for PETG biocarriers. The PP biocarriers showed the lowest density of 0.828 ± 0.014 g cm^−3^, followed by ABS with 0.910 ± 0.003 g cm^−3^. The biocarriers out of ABS and PP showed lower density than the medium and floated on top, while those out of PLA and PETG sank to the bottom during submerged shaking flask cultivation. The optimized PAR biocarrier achieved a density of 1.081 ± 0.017 g cm^−3^ due to the internal air pocket and was thus subject to minimal static buoyancy.

### Wettability and surface roughness of 3D-printed samples

The measurement of contact angle offers information on the wettability of 3D-printed polymers and the influence of different top layer orientations. The results for contact angle are shown in Table 1. Depending on the orientation of the top layer, contact angles of untreated samples varied from 79.3° to 94.5° for ABS, 64.9° to 85.6° for PETG, 70.0° to 97.1° for PLA, and 77.0° to 115.2° for PP. The highest contact angles occurred on transversal orientation for all untreated materials. Contact angle and thus hydrophobicity within constant line orientation significantly decreased due to fluorination for all materials (LSD: *p* < 0.01), varying from a reduction of − 4.3° (PETG, concentric) to − 40.2° (PP, transversal). Fluorinated samples varied in contact angle and values ranged from 54.9° to 64.9° for ABS, 59.6° to 63.8° for PETG, 53.0° to 75.7° for PLA, and 50.1° to 75.0° for PP. The average contact angle across the different top layer orientations was 89.5° ± 16.2° for PP, 84.6° ± 7.4° for ABS, 82.3° ± 11.2° for PLA, and 73.3° ± 11.7° for PETG when untreated respectively 62.4° ± 10.1° for PLA, 61.8° ± 10.6° for PP, 61.4° ± 4.8° for ABS, and 61.0° ± 4.5° for PETG when fluorinated. Based on the average contact angle, among the untreated materials PETG showed significant difference (LSD: *p* < 0.04) and comparison of PP to PLA indicated a trend (LSD: *n* = 12; *t* = 1.7; *p* = 0.091). Other untreated and all fluorinated materials showed no significant variation (LSD: *p* > 0.25).

**Table 1:** Surface properties of 3D-printed samples. Given values represent the mean of three replicates with standard deviation

Top layer orientation	Material	Untreated		Fluorinated	
		Θ (°)	*R*_a_ (µm)	Θ (°)	*R*_a_ (µm)
Diagonal	ABS	82.9 ± 4.6	9.3 ± 3.6	64.9 ± 3.4	3.7 ± 0.8
	PETG	76.7 ± 3.4	7.2 ± 4.4	59.6 ± 3.0	5.2 ± 1.6
	PLA	70.0 ± 2.1	5.2 ± 0.8	53.0 ± 5.2	7.0 ± 0.5
	PP	83.5 ± 3.2	12.7 ± 0.7	60.5 ± 3.2	5.1 ± 0.3
Transversal	ABS	94.5 ± 3.9	1.5 ± 0.7	62.7 ± 3.1	1.7 ± 0.2
	PETG	85.6 ± 13.6	1.0 ± 0.0	63.8 ± 7.5	1.7 ± 1.1
	PLA	97.1 ± 4.8	0.9 ± 0.1	75.7 ± 3.1	1.3 ± 0.3
	PP	115.2 ± 4.7	3.7 ± 1.5	75.0 ± 4.4	2.1 ± 0.5
Longitudinal	ABS	79.3 ± 5.8	13.0 ± 1.1	62.9 ± 5.6	8.0 ± 1.1
	PETG	66.0 ± 4.7	4.7 ± 0.7	59.9 ± 3.2	15.6 ± 3.0
	PLA	78.7 ± 7.0	9.4 ± 1.5	59.2 ± 3.5	8.8 ± 1.2
	PP	82.1 ± 4.5	22.2 ± 1.5	61.7 ± 3.4	20.1 ± 1.6
Concentric	ABS	81.8 ± 2.6	1.6 ± 0.5	54.9 ± 3.4	1.9 ± 0.9
	PETG	64.9 ± 4.8	3.3 ± 1.9	60.6 ± 3.6	1.8 ± 0.9
	PLA	83.5 ± 3.9	2.7 ± 1.6	61.8 ± 8.5	6.1 ± 4.5
	PP	77.0 ± 4.2	3.2 ± 0.4	50.1 ± 8.8	4.8 ± 0.8

*Θ* contact angle, *R*_*a*_ arithmetical mean deviation

Values for surface roughness, represented by arithmetical mean deviation, are shown in Table 1. Surface roughness varied depending on the print orientation of the top layer. Longitudinal and diagonal top layer orientation resulted in rougher surfaces than concentric and transversal orientation. No significant difference (LSD: *p* > 0.2) was observed between concentric and transversal orientation. Fluorination showed no clear effect to surface roughness as values either decreased or increased. Alteration in arithmetical mean deviation through fluorination varied from − 7.6 µm (PP, diagonal) to + 10.9 µm (PETG, longitudinal).

### Biofilm attachment to polymeric biocarriers

**Fig. 4:**
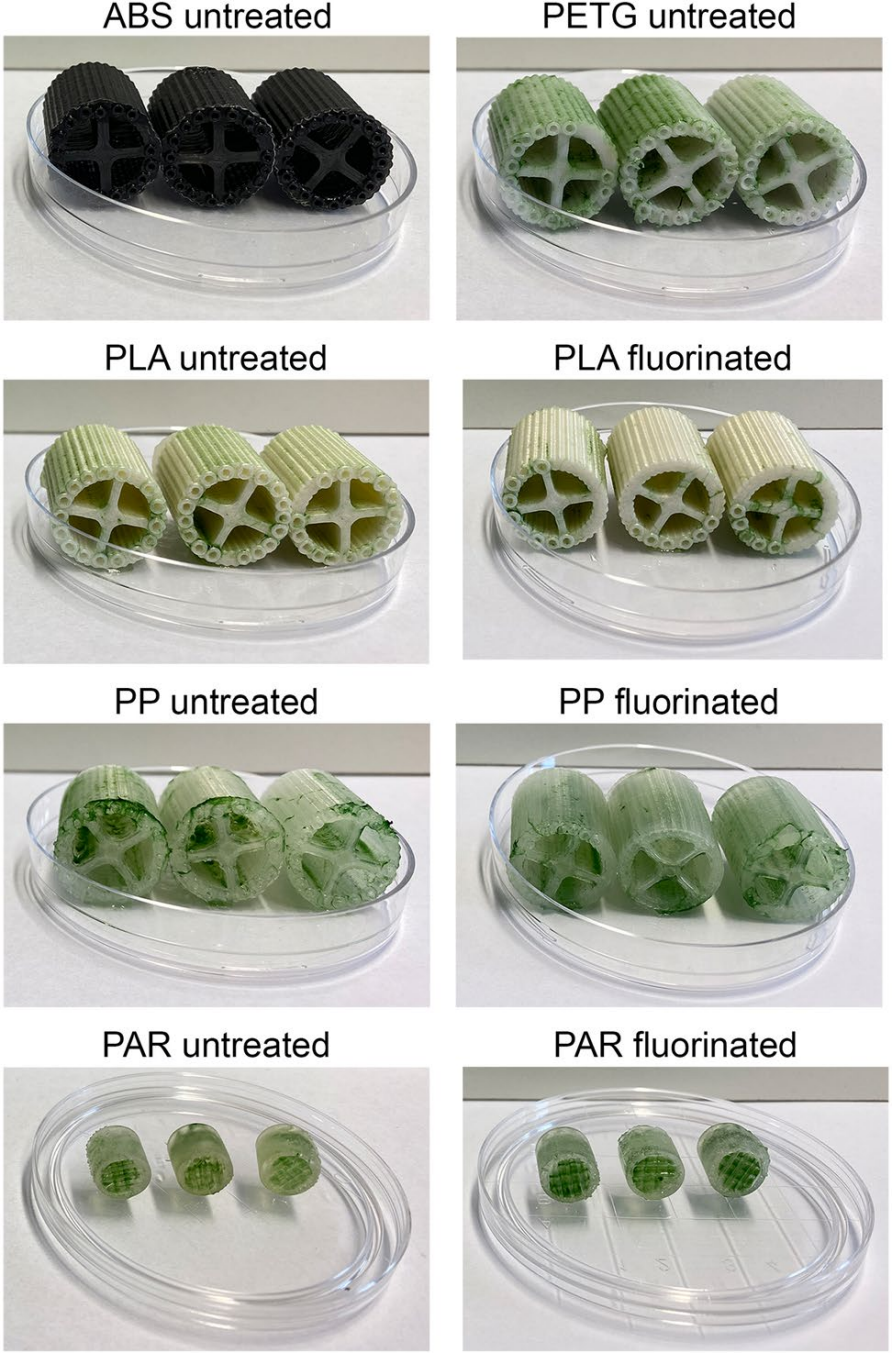
Biofilm distribution on cultivated biocarriers overgrown with *Nostoc flagelliforme* (CCAP 1453/33)

**Fig. 5:**
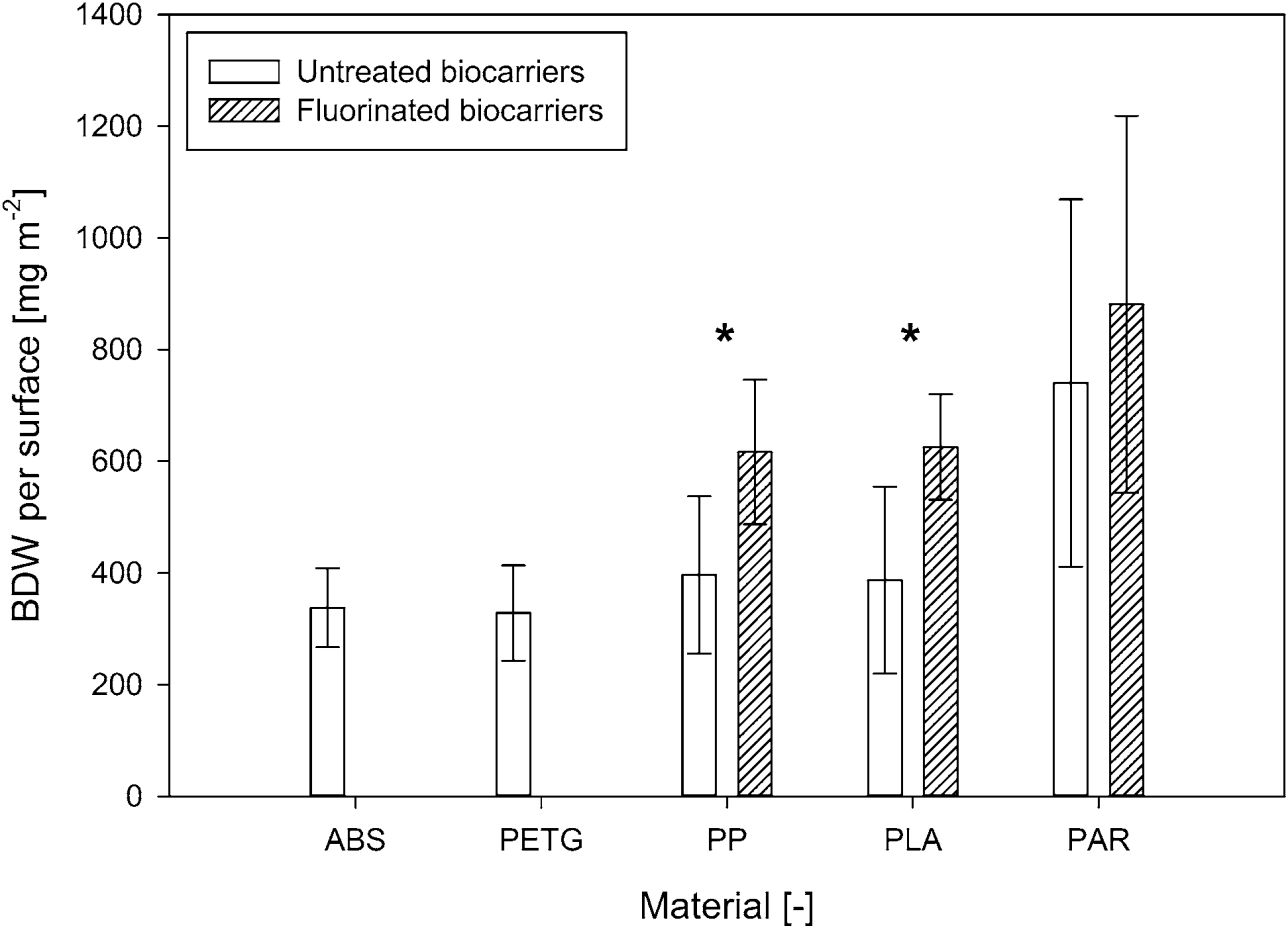
Biomass dry weight (BDW) per biocarrier surface for untreated and fluorinated carriers. Given values represent the mean of 10 biocarriers with standard deviation. * Indicates significant difference in BDW per biocarrier surface between untreated and fluorinated biocarriers for PP (LSD: *n* = 10; *t* = 2.51; *p* = 0.014) and PLA (LSD: *n* = 10; *t* = 2.72; *p* = 0.008)

**Fig. 6:**
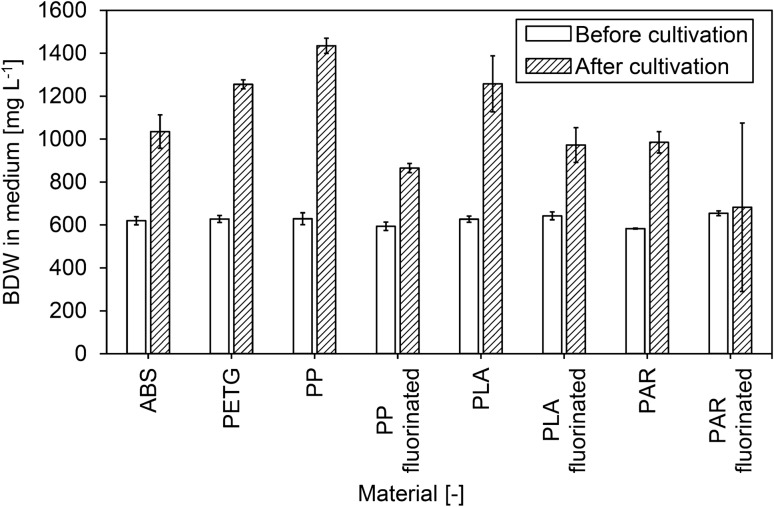
Biomass dry weight (BDW) concentration in the medium before and after cultivation of untreated and fluorinated biocarriers. Given values represent the mean of two flasks per biocarrier type and treatment with standard deviation

The influence of biocarrier materials on the initial biofilm adhesion to different polymeric surfaces was further investigated in submerged cultivation of *N. flagelliforme* (CCAP 1453/33). In addition to the geometrically identical FFF biocarriers (out of ABS, PETG, PP, and PLA), optimized PAR biocarriers were also added to the shaking flask cultivation. After 3 weeks, the attached cyanobacterial biofilm (Fig. 4) was determined gravimetrically for each individual carrier. The BDW of the attached cyanobacteria in relation to carrier surface is shown in Fig. 5. For biocarriers made of ABS, PETG, PP, and PLA, the BDW per biocarrier surface of the untreated biocarriers were in the range of 328.5 ± 85.4 mg_BDW_ m^−2^ to 396.6 ± 140.7 mg_BDW_ m^−2^. Therefore, on the geometrically identical, untreated biocarriers, no significant influence of the material on biofilm formation of cyanobacteria (LSD: *p* > 0.4) was observed. In comparison with these four biocarriers, the adhesion of cyanobacteria on optimized PAR biocarriers resulted in significantly increased BDW per surface (LSD: *p* < 0.001) with 740.1 ± 329 mg_BDW_ m^−2^. Fluorinated biocarriers out of PP and PLA showed significant higher BDW per surface than their untreated counterparts (LSD: *p* < 0.05), while PAR biocarriers revealed only a trend (LSD: *n* = 10; *t* = 1.61; *p* = 0.112). Thus, PP and PLA biocarriers revealed more than 50% increased BDW per biocarrier surface due to fluorination. With a range of 616.6 ± 129.4 mg_BDW_ m^−2^ to 625.9 ± 94.4 mg_BDW_ m^−2^, no considerable difference between fluorinated PP and PLA biocarriers could be measured. The suspended biomass surrounding the biocarriers also increased during cultivation. The remaining BDW in suspension after removal of the biocarriers is shown in Fig. 6. Compared to untreated biocarriers, the fluorinated biocarriers as well as the optimized PAR biocarriers showed less increase in suspended biomass, which corresponds to the increased biomass adhesion observed on the surfaces of these biocarriers. In summary, untreated biocarrier materials did not show any impact on biofilm adhesion of *N. flagelliforme* (CCAP 1453/33) while fluorination and biocarrier design contributed to enhanced biofilm attachment.

## Discussion

### Surface properties of 3D-printed materials

**Fig. 7:**
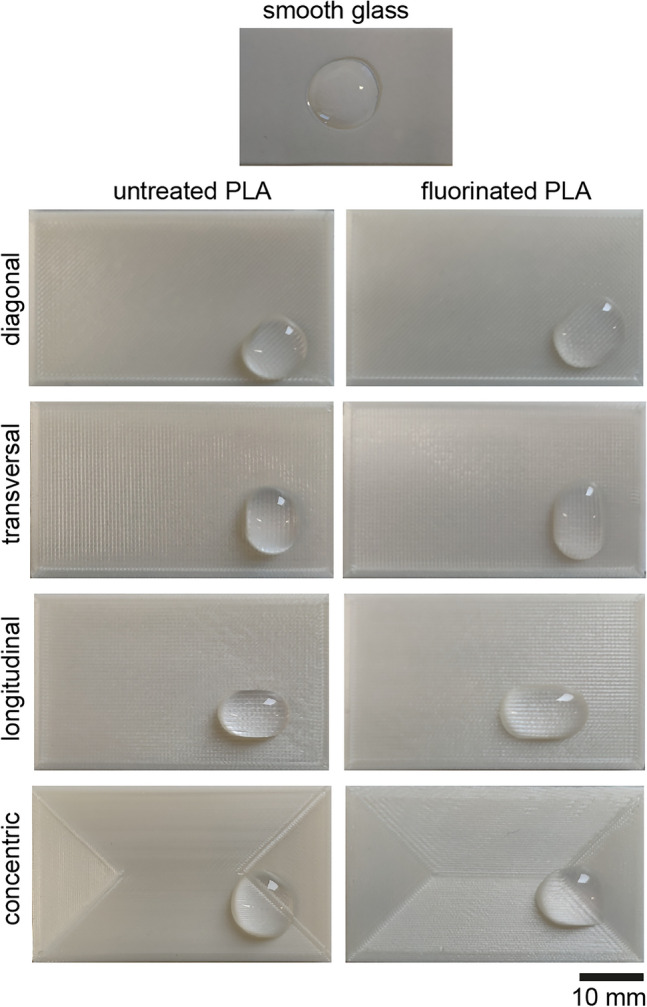
Top view of the drop formation of 300 µL BG 11 drops on smooth glass, without deformation, and on the surface of untreated and fluorinated PLA samples, with deformation in direction of the top layer lines

Wettability directly corresponds to contact angle. Generally, lower contact angles indicate a better wetting and more hydrophilic surfaces, while higher contact angles represent poor wetting and more hydrophobic surfaces. Measurement of contact angles showed that the grooves on the surface of FFF printed material samples influenced drop formation and led to an elliptical deformation in direction of the line pattern as illustrated in Fig. 7 for PLA. The deformation effect occurred on all tested FFF printed surfaces and was even enhanced through fluorination. As the line patterns were oriented differently in relation to the camera view on the samples for contact angle measurements (Fig. 3), the deformation effect resulted in variation of drop width and height and thus caused varying contact angles for the different top layer orientations. Complex geometries like biocarriers resulted in a 3D-printed object with surface patterns that consisted of a mixture of the analyzed line orientations. Due to the different line orientations, varying contact angles on the active surface of the biocarriers are to be expected. Therefore, no general contact angle of the biocarrier could be given. To characterize the surface properties, the average contact angles were determined at four different line orientations.

For PLA, the average contact angle of BG 11 medium was 82.3° for untreated and 62.4° for fluorinated samples. This is close to the water contact angle of 81.1° for untreated PLA and 61.0° for fluorinated PLA observed in a similar fluorination process [31].

Surface roughness of the analyzed samples was comparable to each other and depended on the line orientation of the top layer. The number of grooves on the surface, caused by FFF 3D-printing, related to the differentiation in surface roughness. Fluorination resulted in either reduction or increase of surface roughness. The highest alteration of arithmetical mean deviation by fluorination was approximately 10 µm. Since individual samples were used for untreated and fluorinated measurements, their roughness difference could also be caused by the fabrication process as the alteration lies well within the expected accuracy of FFF printing. Therefore, it is not clear that differences in surface roughness between untreated and treated samples originate from the fluorination process. However, it is assumed that fluorination does not alter surface roughness, which corresponds to the observations by Schroepfer et al. [31].

### Influence of wettability and surface roughness on biofilm adhesion

**Fig. 8:**
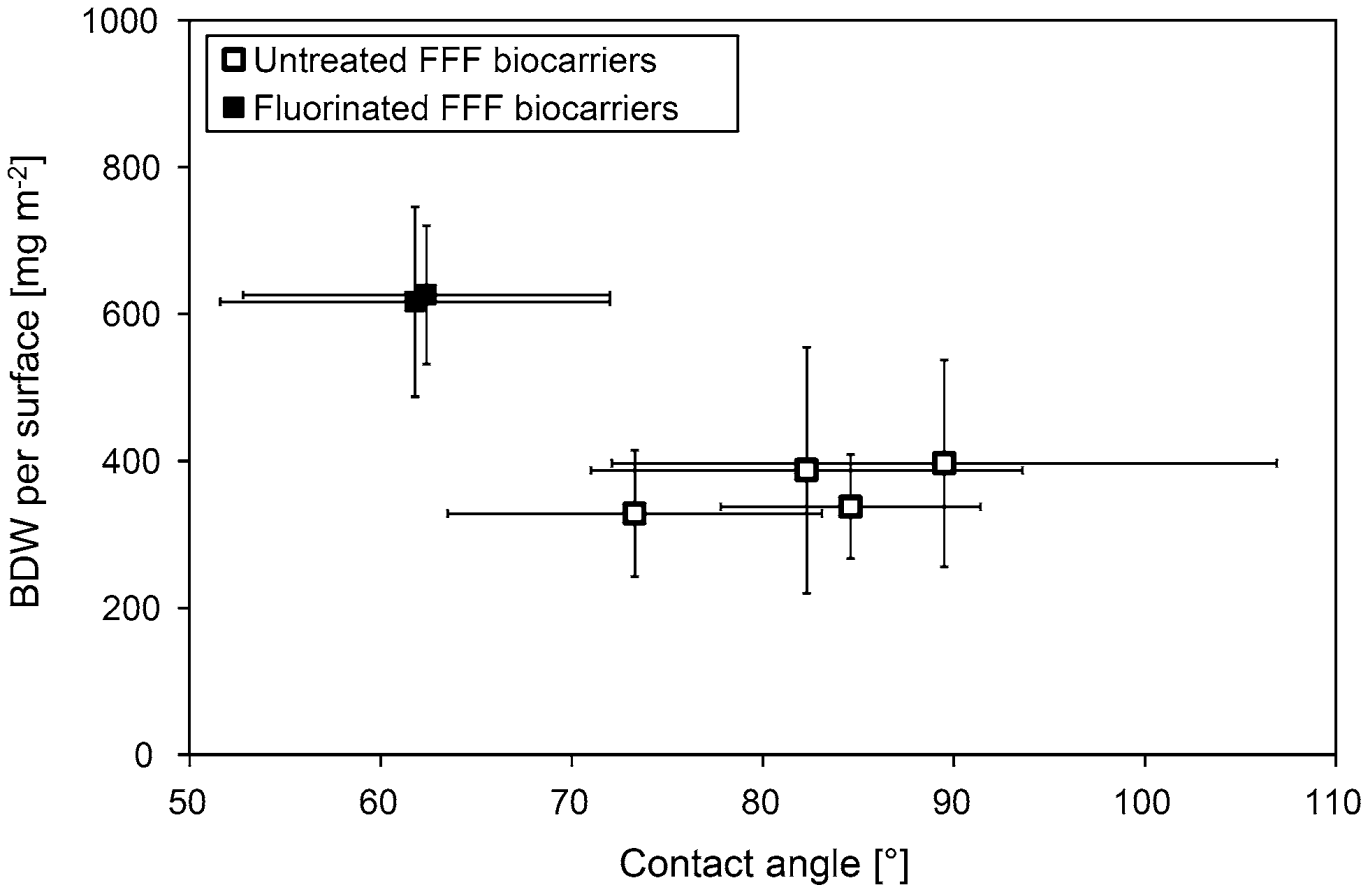
Biomass dry weight (BDW) per biocarrier surface in relation to average contact angle for untreated and fluorinated FFF biocarriers. Given values represent the mean of 10 biocarriers and the average material contact angle for BG 11 media with standard deviation

The influence of wettability on biofilm adhesion is a highly discussed topic. Apart from various biofilms investigated, in most studies several materials have been used to achieve different wettability for the investigation of biofilm adhesion. Material composition also influences biofilm adhesion [27], which makes it difficult to isolate the effect of wettability. In this study gas-phase fluorination showed a significant reduction of contact angle and thus caused an alteration of the wettability for all materials. Although the chemical composition of the materials was also modified through fluorination, this resulted in different wettabilities for the same material and simplified the comparison and isolation of the influence of wettability. The correlation between attached biomass and the average contact angle of the biocarrier materials is shown in Fig. 8. Based on the investigation of the surface properties between the untreated FFF printed biocarriers (ABS, PETG, PLA, and PP), there was no considerable difference found in biofilm adhesion, which corresponded to the similar wettability observed for the tested materials. Fluorination however resulted in a significant reduction of the average contact angle of approximately 20° for PLA and 28° for PP. For all investigated biocarriers, fluorination increased wettability and hydrophilicity while the surface roughness remained almost unaltered. The trend of higher biomass attachment on fluorinated biocarriers of the same material, compared to untreated carriers, indicated that biofilm attachment by *N. flagelliforme* (CCAP 1453/33) is preferred on hydrophilic surfaces with high wettability. Several other studies reported enhanced biofilm adhesion to hydrophobic surfaces with low wettability [27, 32–34], no influence of wettability to biofilm adhesion [35] as well as increased adhesion to hydrophilic surfaces with high wettability [36]. The different findings relate to different species such as algae and cyanobacteria [27, 32, 34, 35], macroalgae [33] and active sludge in wastewater treatment [36]. The diversity in these findings indicates that the preferred surface characteristic (hydrophobic or hydrophilic) highly depends on the used microorganism and that the results are not directly transferable to other cyanobacteria species. Furthermore, there are several reports for the same microalgae species (*Chlorella vulgaris)* describing enhanced adhesion to hydrophobic surfaces [27, 32] and no influence of hydrophobicity on biofilm adhesion at all [35]. This indicates, although wettability has a major influence on the initial biofilm formation, that other factors such as adaptation of the microorganisms [35], media composition [32], material compatibility [27], or cultivation conditions are also crucial and make it difficult to generalize the prediction of biofilm formation.

In this study, the surface roughness was measured primarily to show that the fluorination process does not have a significant impact on surface roughness. The observed changes in roughness were rather small, occurred randomly, and most likely due to inaccuracies in the manufacturing process. The influence of surface structure and roughness on biofilm adhesion has been previously studied by other researchers [28, 29, 36–39]. It is reported that surface features of similar size to the individual cells in the biofilm are beneficial for surface adhesion, while smaller features decrease biofilm adhesion [37]. Furthermore, the optimal surface roughness highly depends on the used microorganisms [29]. However, in terms of biocarriers, several findings report increased biomass adhesion to rougher surfaces [28, 36, 39] due to protection of cells from hydrodynamic forces [38] and an increased effective surface due to microscopic surface features [28]. Apart from surface roughness, biocarrier design and dimensions are also important for the initial biofilm formation.

### Influence of biocarrier design on biofilm adhesion

Increased biofilm attachment per biocarrier surface on optimized PAR biocarriers showed the importance of biocarrier design for biofilm formation and growth. The immobilization of biomass on biocarriers depends not only on surface properties like wettability and surface roughness, but also on hydrophobicity of the microorganisms, electrophoretic mobility and steric effects [40, 41]. Hydrodynamic forces also play a key role for the initial biofilm formation on the biocarriers. For the determination of biofilm attachment in this study, a static cultivation was performed to reduce the risk of biomass detachment from the carriers. In static cultivation, sedimentation reduces the amount of biomass in suspension and can lead to poor biofilm formation because less biomass is available for attachment. This is supported by the low amount of biomass on the biocarriers observed in our experiments. However, mixing during cultivation results in increased shear forces that could lead to detachment of biomass from the biocarrier surfaces. To prevent biofilm detachment by erosion due to fluid shear forces, abrasion due to collision with other particles, or sloughing [42], biocarriers should have recessed areas to protect the biomass from shear forces. Furthermore, optimized biocarriers should have a highly effective contact area to facilitate biomass adhesion and a high loading capacity to ease the diffusion of nutrients [25, 42]. A biocarrier density similar to the medium density reduces hydrostatic forces and is expected to ensure optimal flow and homogeneous biofilm attachment around the biocarrier in submerged cultivations. Because inoculation for emerged cultivations is usually carried out in suspension, a neutrally buoyant biocarrier could also ensure uniform biofilm inoculation before emerged cultivation.

Regarding different biocarrier structures, the highest growth of heterotrophic and phototrophic biofilms has often been observed in the protected internal structures and cavities when used in fluidized beds [36, 43, 44]. As the cyanobacteria tended to adhere to the grooves of the lateral area on the cultivated biocarriers (Fig. 4), it is assumed that cyanobacteria also prefer protected areas for initial biofilm formation in static cultivation.

Although the internal areas provide good protection against shear forces, light transmitting into these areas can be affected by several factors. Biomass on the outer shell of the biocarrier, adjacent biocarriers and suspended cells surrounding the biocarriers [45] reduce light penetration and thereby affect photosynthesis and biomass production in the protected internal structures. However, cyanobacteria and especially immobilized terrestrial cyanobacteria are highly adaptable to extreme and fluctuating light conditions, which is shown by their natural occurrence in harsh environments like the Antarctica [46], tropical regions [47], or deserts [48]. Light acclimation and adaptation enables cyanobacteria to produce biomass under extremely low light intensities [49] and even increases production of certain valuable pigments like chlorophyll, phycobilisomes or specific carotenoids [50]. The biomass in the internal structures of the biocarriers is expected to continue to grow due to light adaptability of cyanobacteria, even if most of the light is blocked by biofilms on the outer shell or other biocarriers. Apart from the biocarrier design, material transparency and color are also expected to show an effect on light availability and biofilm growth [51]. However, further investigations are needed to clarify the impact of the light limitation on biofilm production in the internal structures.

In conclusion, no significant differences of biomass adhesion were observed on the tested untreated biocarrier materials. In contrast, fluorination, particularly by PP and PLA biocarriers, showed significant improvement in biofilm formation because of increased wettability. The higher biofilm attachment per carrier surface to optimized PAR biocarriers revealed great potential for further optimization of biocarrier geometry and thus biotechnological production of phototrophic biofilms.
